# A renewed focus on carbon uptake and use to enhance crop performance in the face of rising temperatures

**DOI:** 10.1093/plphys/kiag548

**Published:** 2026-07-27

**Authors:** Andrew P Scafaro, Onoriode Coast, William T Salter, Nicolas L Taylor, Rebecca J Thistlethwaite, Daniel Cowan-Turner, Owen K Atkin

**Affiliations:** Division of Plant Sciences, Research School of Biology, Australian National University, Canberra, ACT 2601, Australia; School of Environmental and Rural Sciences, Faculty of Science, Agriculture, Business, and Law, University of New England, Armidale, NSW 2350, Australia; The University of Sydney, Plant Breeding Institute, School of Life and Environmental Sciences, Narrabri, NSW 2390, Australia; School of Molecular Science, Australian Plant Phenomics Network, ARC Training Centre in Predictive Breeding for Agricultural Futures and UWA Institute of Agriculture, The University of Western Australia, 35 Stirling Highway, Crawley, Perth, WA 6009, Australia; The University of Sydney, Plant Breeding Institute, School of Life and Environmental Sciences, Narrabri, NSW 2390, Australia; Division of Plant Sciences, Research School of Biology, Australian National University, Canberra, ACT 2601, Australia; Division of Plant Sciences, Research School of Biology, Australian National University, Canberra, ACT 2601, Australia

## Abstract

Increasing crop production to meet global food demand amid a more unstable climate is a challenge that requires new approaches from science and technology. To meet this challenge, plant physiologists must coordinate efforts with crop breeders to select appropriate traits and associated genes that drive improvements in crop performance. However, improvements in yield gain and yield quality are often blind to mechanistic understanding. Recent insights suggest that a deeper understanding of carbon metabolism could contribute to step changes in the yield potential of major crops. By enhancing the efficiency of turning captured light and carbon into biomass via increasing photosynthesis and the efficiency of respiratory energy production and use, crop yields could be dramatically enhanced. Exploiting this opportunity requires the technical ability to identify germplasm with desired carbon metabolism traits, because such traits are especially responsive to developmental changes, tissue type, and environmental perturbations. For practical field-based selection for beneficial performance in carbon metabolism, physiologists must broaden their experimental procedures to incorporate the temporal and spatial dynamics of carbon capture and its use. Advancements in remote sensing and artificial intelligence may prove crucial on this front, launching a new path to crop improvement.

## Introduction

**Figure 1:**
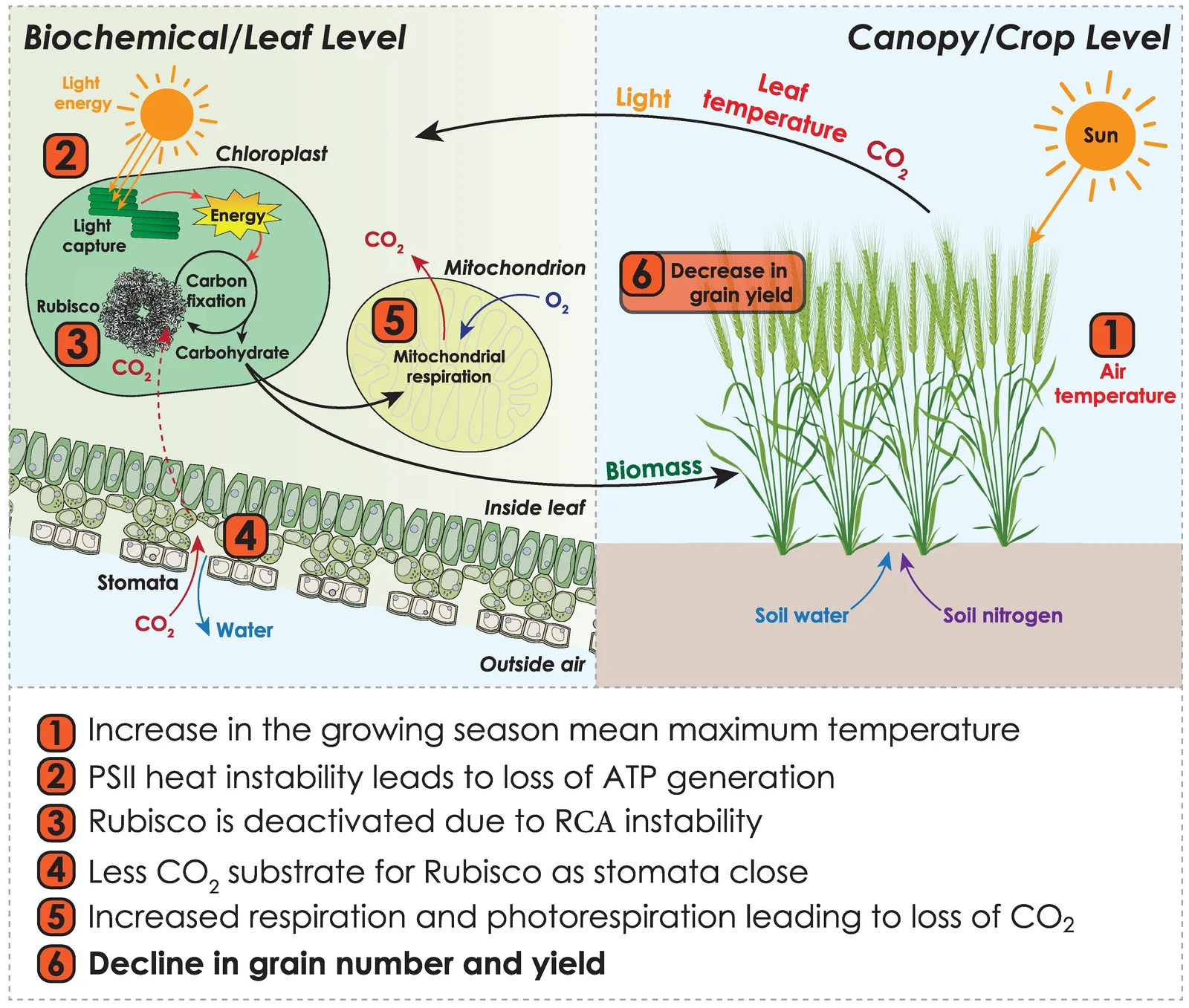
Schematic outlining the physiological effects heat stress can have on crops and how an increase in average air temperature during the growing season is likely to reduce biomass accumulation and yield. Abbreviations: PSII, photosystem II; RCA, Rubisco activase.

Based on current trajectories, food demand will increase by 50% to 60% in the first half of this century, driven mostly by population and income growth (van Dijk et al. 2021; Falcon et al. 2022). Yet yields of staple crops like maize (*Zea mays*), rice (*Oryza sativa*), wheat (*Triticum aestivum*), and soybean (*Glycine max*) have historically only been increasing by 0.9% to 1.6% per year (Ray et al. 2013). Impeding efforts to increase yield are increasingly unstable environmental conditions, especially with rising global temperatures (Peng et al. 2004; Lobell and Gourdji 2012; Vogel et al. 2019). Figure 1 provides an example of the heat-susceptible aspects of leaf physiological processes that have a detrimental impact on crop yields as global temperatures rise (detailed in previous reviews [Wahid et al. 2007; Slattery and Ort 2019]). Central to the way heat affects crop growth is its impact on photosynthetic carbon uptake and respiratory carbon release, with heat stress reducing biomass accumulation during vegetative growth and subsequent investment in grain yield. The growing gap between heat-induced losses in crop yields and the rising demand for food will widen in the coming decades unless science and technology develop industry-relevant solutions that minimize heat-induced reductions in net carbon uptake by crop canopies.

A commentary article by Khaipho-Burch et al. (2023) emphasized the 5 key criteria that should be the focus of plant science in tackling crop yield questions: (i) clearly define the yield; (ii) replicate experiments at the field level across geographical locations and years; (iii) use plant material and growing setup that match those of farming; (iv) include appropriate controls, such as the most current elite-performing lines, to validate performance; and (v) prioritize genes that plant breeders do not already focus on. In this review, criterion 1 is detailed using defined yield equations, while criteria 2 to 4 are examined from the perspective of emerging technologies that can integrate experiments spatially and temporally in real-world farming environments. The fifth criterion relating to selecting novel genes is crucial, and fortunately, plant physiologists have a strong understanding of the role of carbon exchange in determining rates of biomass acquisition and plant performance (Long et al. 2006; De Block and Van Lijsebettens 2011; Evans 2013; Croce et al. 2024; Wu et al. 2024). Biomass is derived through net photosynthetic carbon assimilation (*A*_net_) and dark respiratory CO_2_ release (*R*_dark_). The recognition of the importance of *A*_net_ and *R*_dark_ and recent technological developments enabling large-scale screening of both traits (temporally and spatially) (Silva-Perez et al. 2018; Coast et al. 2019; Joshi et al. 2023; Amthor 2025; Gaju et al. 2025) provide an opportunity for breakthroughs in yield potential to be achieved through a renewed focus on time-integrated carbon uptake and efficiency of use.

Physiologists pair measurements of leaf *A*_net_ and *R*_dark_ through gas-exchange instruments with photosynthesis theory (Farquhar et al. 1980; von Caemmerer and Farquhar 1981). This has enabled insights into carbon metabolism and its influence on plant performance from the leaf to the ecosystem level (von Caemmerer 2020). However, studies most often focus on rates of carbon flux in stable environments, termed steady-state photosynthesis (ie, at a set irradiance, temperature, and CO_2_ concentration) (Long and Bernacchi 2003). Moreover, physiologists usually take one set of measurements on a distinct tissue type (most often a fully expanded, mature leaf) at a defined point in the plant's life to determine characteristics of photosynthetic performance, such as the maximum rate of ribulose-1,5-bisphosphate carboxylase/oxygenase (Rubisco) CO_2_-fixing capacity determined from gas exchange measurements (*V*_cmax_). While such experiments provide valuable insights into a plant's photosynthetic and respiratory characteristics and the limitations that set performance, they often correlate poorly with final biomass and crop yields (Driever et al. 2014). This is because biomass and crop yield integrate both carbon uptake and its efficiency of use over the entire life of a crop (Zelitch 1982) and across foliar and nonfoliar tissues. Ultimately, yield depends on whole-plant physiological performance over time, developmental progression, genetics, and responses to husbandry and the growth environment.

The lack of correlation between yield and single-point measurements of *A*_net_ and *R*_dark_ does not imply that no relationship exists; rather, it reflects the disconnect between static physiological assays and the integrated, dynamic nature of crop performance over time in the field. To establish this linkage, physiologists can broaden mechanistic insights by examining how carbon metabolism operates under real-world agricultural conditions. That is, rather than isolating steady-state processes in controlled settings, physiologists should investigate *A*_net_ and *R*_dark_ across diurnal and seasonal timescales, under the fluctuating light, temperature, and water availability conditions experienced in the field. In addition, physiologists can (i) work at the whole-plant and canopy scales; (ii) prioritize trait integration over the crop life cycle; (iii) account for contributions from non-foliar tissues such as stems, tillers, spikes and roots, plant parts that can significantly influence final yield but are often overlooked in leaf-centric studies; and (iv) evaluate physiological performance in field trials with elite cultivars under representative management. This would also generate time-resolved context-rich data for improved parameterization and validation of process-based crop models. More recent carbon capture and use models of crop performance that explicitly account for environmental perturbations over a crop's life cycle can more accurately predict yield (Wu et al. 2019; Wu et al. 2023).

This review is centered on carbon metabolism as a lever for yield improvement. Section 2 demonstrates that yield potential can be enhanced through gains in radiation use efficiency (RUE), which hinges on improving net photosynthesis (*A*_net_) and the efficiency of dark respiration (*R*_dark_). Section 3 details why capturing the full value of these traits requires assessing their temporal dynamics (across diurnal and seasonal scales) and spatial variation (across foliar and non-foliar tissues) under realistic field conditions. Section 4 addresses the experimental challenges of such measurements and the limitations of controlled environments. Finally, Section 5 highlights how advances in sensor technology, remote phenotyping, and machine learning are providing the tools needed to phenotype carbon metabolism at scale through time and space, thereby closing the gap between mechanistic understanding and breeding application.

## Yield potential can be enhanced through gains in radiation use efficiency

Yield is the ultimate indicator of crop performance across variable environments (Duvick 2005; Fischer et al. 2014). Although the mechanisms underpinning yield gains are not fully understood, yield can be quantified using a straightforward physiological framework (Monteith 1977; Reynolds et al. 2012). Yield potential (*Y*_p_), the theoretically maximized yield achieved on a farm and attributable to crop genetics, can be formulated as


(1) $$Y_{p}=Q\times f\;\times \;RUE\;\times HI$$

where *Q* is the cumulative incident solar radiation per unit of ground area throughout the growing season (eg, MJ m^−2^); *f* is the proportion of *Q* intercepted by the canopy; RUE is radiation use efficiency, a measure of how efficiently a crop converts intercepted solar radiation into biomass (eg, g MJ^−1^); and HI is harvest index, the proportion of crop biomass that is harvested.

Importantly, the yield equation assumes that light is the dominant limiting factor for yield under optimal agronomic conditions, with carbon assimilation scaling directly with intercepted radiation (see Box 1 for further discussion of the relationship between *Q* × *f* and RUE). Foulkes et al. (2009) noted that while the equation appears simple, it highlights the complex process of yield formation and the key areas that plant breeders could target to enhance yield. Indeed, there are many potential ways to approach this equation to improve yield. For example, in maize, researchers are exploring the most efficient canopy architecture to enhance light capture and improve the *f* term of the equation (Perez et al. 2019; Lacasa et al. 2022). In recent decades, some physiologists have targeted yield improvements by focusing on enhancing the RUE term in Equation 1 (Long et al. 2006; Reynolds et al. 2012; Evans 2013; Ainsworth and Long 2021; Garcia et al. 2023; Croce et al. 2024).

Box 1. **Light interception considerations of radiation use efficiency**

One interpretation of Equation (1) is that the amount of radiation intercepted by the canopy (ie, Q×f) is independent of radiation use efficiency (RUE). However, RUE is itself radiation dependent. This is because the amount of CO_2_ fixed per unit of radiation captured (α) in the RUE equation is especially dependent on (i) the amount of radiation absorbed by the canopy at any given time; and (ii) the investment of N in photosynthetic capacity due to longer-term sun and shade acclimation. Although not equivalent to an entire canopy, these concepts can be demonstrated using previous observations of light-dependent photosynthesis in top and mid-canopy leaves of crops such as wheat and rice (Townsend et al. 2018; Guo and Lv 2025). Short-term photosynthesic adjustment to light (Box Fig. 1A) demonstrates that the amount of CO_2_ uptake is not proportional to light absorbed (ie, α is a light-dependent variable). N and photosynthetic machinery are partitioned differently based on whether a leaf has adjusted over days to weeks to sun or shade (long-term light acclimation), leading to varying CO_2_ uptake at a given irradiance for top and mid-canopy leaves. At any given irradiance, α is equal to the CO_2_ fixation rate divided by the amount of intercepted irradiance. Multiplying α by a constant biosynthesis efficiency (ε_biosynthesis_) estimates a proxy of RUE at a given value of intercepted irradiance by a leaf (Box Fig. 1B). This analysis demonstrates 2 main points: (i) that RUE declines with rising intercepted irradiance above moderate light levels (∼200 µmol quanta m^−2^ s^−1^) irrespective of longer-term light acclimation and (ii) that top-canopy leaves, which are more exposed to stronger light, have a higher RUE than lower-canopy leaves at and above the irradiance where RUE peaks (Box Fig. 1B).

**Box Figure 1:**
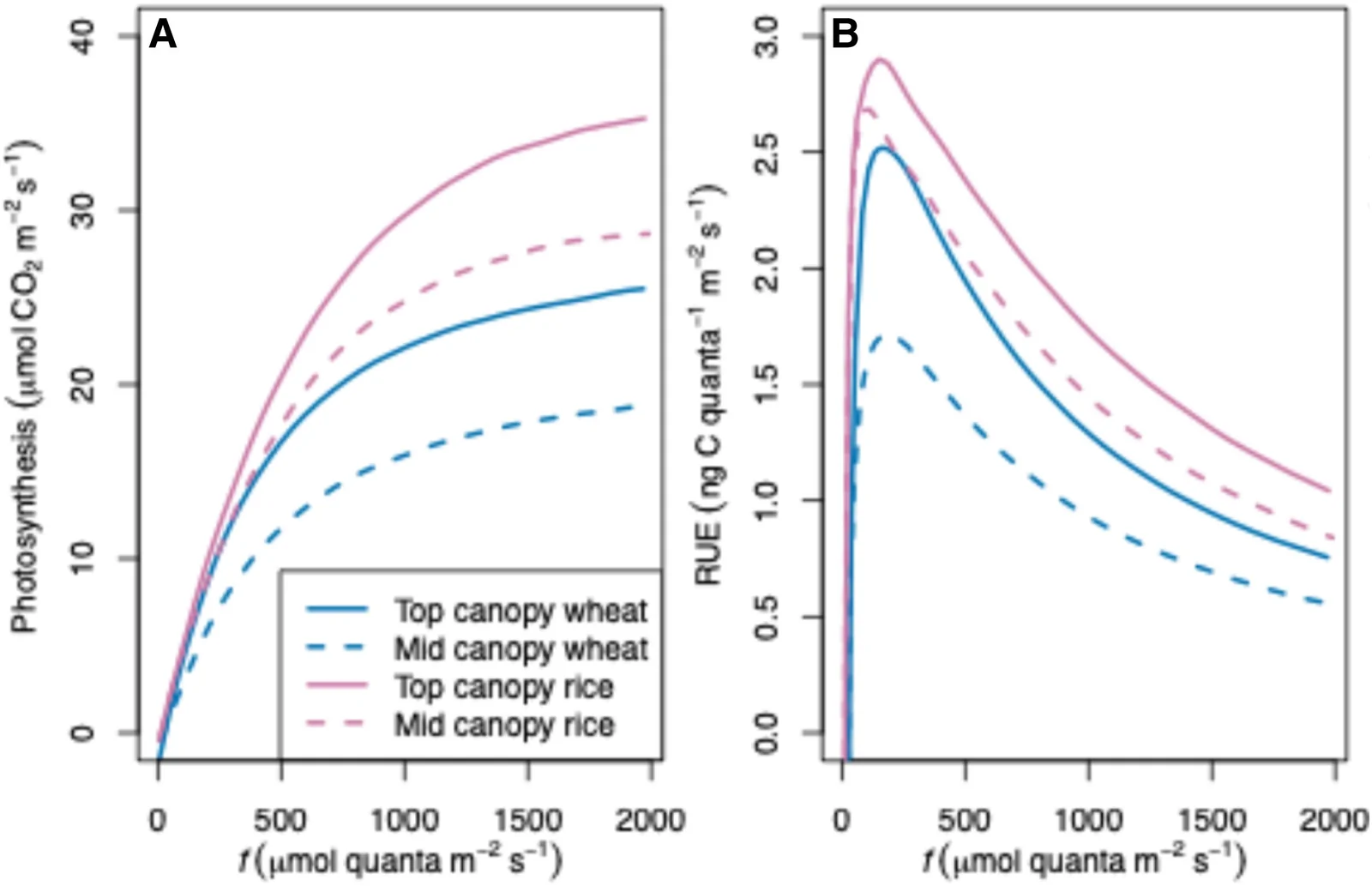
Rates of net photosynthesis and radiation use efficiency (RUE) at a given amount of radiation captured (*f*). (A) The observed instantaneous light response curves of top and mid-canopy leaves from rice and wheat based on the reports of Guo and Lv (2025) and Townsend et al. (2018). (B) RUE was calculated by factoring in the amount of photosynthetic C per unit of *f*, and assuming a fixed biosynthesis efficiency (ε_biosynthesis_) of 0.7 C retained per C fixed.

When modeling the impacts of improvements in RUE on yield, it is assumed that (i) the crop is limited by carbon supply and not by sink strength and (ii) RUE is largely independent of other terms in the yield equation, namely incident radiation received and captured (*Q*  *×*  *f*). Support for a key component of the first assumption, that being that productivity is not limited by sink strength, comes from the fact that carbon investment in harvestable tissue (ie, the HI) has already been fully exploited. This is evident in HI values, which usually range from 0.4 to 0.6 in most crops, including cereals like wheat and rice, grown in resource-rich environments (ie, well-fertilized and irrigated fields) and selectively bred to maximize their HI (Hay 1995). Given that harvestable tissues represent strong sinks for fixed carbon, maximizing HI means that breeders will have selected for strong sink demand. Despite this, source and sink traits are both important contributors to improved crop yield in dynamic field environments, even in high HI crops like wheat (Wang et al. 2026). Within the scope of this study, we focus on source traits that maximize carbon uptake and are yet to be fully optimized.

With respect to the second assumption, it cannot be assumed that RUE is independent of the incident radiation received and captured. The reason for this is that RUE scales with *Q*  *×*  *f* until a point at which *Q*  *×*  *f* saturates the photosynthetic machinery of a leaf (Wilson et al. 1992), illustrated in Box 1. This interaction between the instantaneous amount of light received and RUE is further influenced by whether a leaf is more sun-acclimated in the top canopy or shade-acclimated lower in the canopy (Box 1). While a single leaf is not a canopy, cross-scale models can be used to derive biomass production by integrating individual leaf RUE performance at varying levels of the canopy into an entire crop performance (Wu et al. 2023, 2024).

Notwithstanding the possible interaction between RUE and irradiance, theoretically, RUE has the potential to be doubled (Long et al. 2006; Garcia et al. 2023) through improvements in the amount of CO_2_ fixed per photon captured (α) and/or through improvements in biosynthesis efficiency (ε_biosynthesis_), the efficiency with which crops accumulate biomass per unit of CO_2_ fixed (g biomass/g CO_2_ fixed) according to Garcia et al. (2023):


(2) $$RUE=\alpha \times \varepsilon_{biosynthesis}$$

In turn, ε*_biosynthesis_* is the product of 2 factors: (i) the efficiency of respiratory ATP synthesis (ε_prod_, mol ATP/g CO_2_ fixed) and (ii) the efficiency with which respiratory ATP is used to drive biosynthetic, transport, and maintenance processes (ε_use_, g biomass/mol ATP). As a result, RUE is related to *α*, ε_prod_ and ε_use_ (Garcia et al. 2023):


(3) $$RUE=\alpha \times \varepsilon_{\;prod}\times \varepsilon_{use}$$

While it is not yet possible to directly measure all parameters in Equation 3, parameter estimates and targets for optimizing energy production and use are outlined in detail in the review by Garcia et al. (2023). For example, improved stress resistance that minimizes the alternative oxidase and uncoupling pathways—pathways that dissipate the electrochemical gradient of mitochondria as heat rather than ATP production—would improve ε_prod_. An example of how ε_use_ can be a target for RUE improvement is through efficient transporter proteins, their abundance, and suitability to the prevailing environment (eg, soil pH, N availability) that can lead to less ATP consumption for matching phloem loading and nutrient uptake. On a broader scale, our knowledge of plant physiology (von Caemmerer and Farquhar 1981), empirical modeling (von Caemmerer 2000; Wu et al. 2019) and proximal remote sensing (Silva-Perez et al. 2018) provide approaches to estimate variations in α and ε_biosynthesis_ in field settings. Ultimately, the goal is to maximize *A*_net_ and its use in building biomass while minimizing inefficient *R*_dark_, noting that *R*_dark_ is the least explored aspect of the conventional yield equation.

## Photosynthetic and respiration efficiency as a means of enhancing crop performance

**Figure 2:**
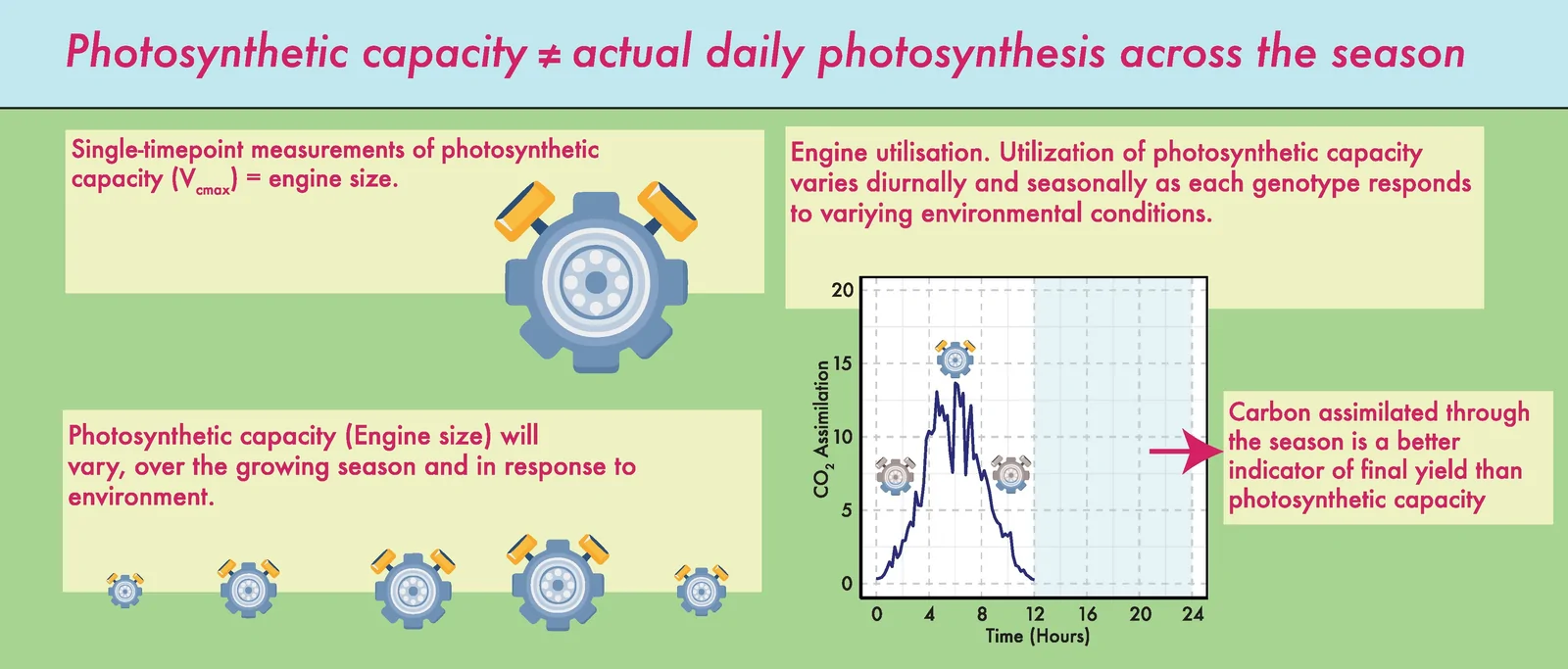
Schematic outlining the limitations of comparing plant photosynthetic performance by single-time-point measurements of photosynthetic capacity (depicted pictorially as different-sized engines), which fail to capture seasonal changes in capacity or diurnal variations in its utilization. These dynamics are driven by genotype-specific responses to environmental conditions, which is why single measurements of photosynthetic capacity often correlate poorly with yield. Measurements of carbon assimilated through the season, or modeling based on genotype-specific photosynthetic responses to the environment, would better link to yield than single-time-point measurements of capacity.

Photosynthetic capacity, evident in *V*_cmax_, correlates with N, as a strong relationship exists between the investment of a leaf in photosynthetic machinery and the N concentration of a leaf (Evans 1989; Kattge et al. 2009; Evans and Clarke 2019). The link between N, photosynthetic performance, and yield exists in crops such as wheat and rice, explaining why N fertilization can dramatically increase yield (Makino 2011). While a focus on photosynthetic capacity is relevant for efforts to improve crop yields, ultimately it is the rates of achieved *A*_net_ in the field, often under environmental constraints, that determine biomass accumulation and contribute to yield. As a result, technologies for large-scale screening for variation in achieved rates of *A*_net_ under field conditions are needed to evaluate links between photosynthetic metabolism and yield potential (Fig. 2). A prime example of an environmental constraint on *A*_net_ is water supply, which strongly affects photosynthesis by influencing stomatal conductance (*g*_s_). Water deficit-induced reduction in *g*_s_ limits the supply of CO_2_ at the site of fixation. Empirically based predictions exist to account for the response of *g*_s_ to the environment (Collatz et al. 1991; Lin et al. 2015), but its complex links to soil water availability and the vapor pressure deficit of the surrounding air (Grossiord et al. 2020) add complexity when attempting to derive *A*_net_ from photosynthetic capacity traits. Adding to *g*_s_ complexity is the emerging realization that rising temperatures can decouple *g*_s_ from *A*_net_, as *g*_s_ plays an important secondary role in cooling leaves through transpiration, irrespective of water loss and photosynthetic requirements (Urban et al. 2017; Aparecido et al. 2020; Diao et al. 2024). The implications of water availability and leaf temperature for *g*_s_ complicate the relationship between *V*_cmax_ and *A*_net_.

Rates of leaf *R*_dark_ are much slower than *A*_net_, especially in fast-growing crops. Indeed, at saturating light and temperatures below 30 °C, *R*_dark_ usually reaches less than 5% of the rate of *A*_net_ (Drake et al. 2016; Rashid et al. 2020a). However, leaf *R*_dark_ continues throughout the night and occurs in all plant cells, resulting in 20% to 40% of photosynthetic carbon gain being released back to the atmosphere and foregone as biomass (Atkin et al. 2007; Amthor 2025). Thus, improving the efficiency of leaf *R*_dark_ may lead to large savings in foregone carbon that could otherwise contribute to increased biomass. Efficiency could be achieved through improvements in respiratory ATP generation and ATP use. Hauben et al. (2009) reported that selection of slower, more efficient respiring canola genotypes across multiple generations was associated with higher yields. Thus, there is scope for yield improvements by screening for variation in *R*_dark_ and its relationship with *A*_net_ and growth to select for high carbon use efficiency rather than low rates of *R*_dark_ per se.

Identifying the heritability of *R*_dark_ in crops is a must if it is to be accurately targeted for breeding. In well-planned, high-throughput experiments with sufficient statistical power to accurately partition sources of variance, it is possible to determine the extent to which genotype contributes to energy metabolism traits that are heavily influenced by the environment (Moshelion et al. 2024). A recent study in wheat grown in environmentally heterogeneous field trials found that 60% of the variation in *R*_dark_ was attributable to the environment and 30% to genetics (Gaju et al. 2025). In the study by Gaju et al. (2024), application of a high-throughput method of estimating *R*_dark_ (Scafaro et al. 2017a) resulted in a dataset comprising over 5,800 measurements of *R*_dark_, with analysis revealing that unexplained variance was reduced to less than 10%, a marked improvement over past studies where unexplained variance for leaf respiration was often greater than 60% (Turnbull et al. 2016; Bloomfield et al. 2018). The previous example demonstrates that traits like *R*_dark_ (and similarly *A*_net_) that are environmentally sensitive can be characterized and selected for in the field through well-designed experiments using high-throughput technology.

The importance of respiratory carbon loss becomes starker as temperatures rise, because *R*_dark_ increases faster than *A*_net_. More than 20% of light-saturated *A*_net_ is immediately respired by leaves when temperatures exceed 40 °C (Drake et al. 2016; Rashid et al. 2020a). The importance of leaf *R*_dark_ as a contributor to crop performance, especially in relation to rising temperature, is evident in the negative impact of night rather than day warming on crops. Rice grown in fields across Southeast Asia showed a strong negative correlation between yield and biomass with a 1.13 °C rise in night temperatures from 1979 to 2003, but no discernible impact on these traits with a 0.35 °C concurrent warming of days (Peng et al. 2004). Thus, improving the efficiency of respiratory metabolism has the potential to significantly reduce carbon loss, especially considering that night temperatures, more so than day temperatures, are warming with climate change (Davy et al. 2017). This fact highlights the need to focus on leaf *R*_dark_ processes despite their complex nature.

A recent characterization of respiratory costs in crops has found that growth and maintenance costs are essentially equally split in their contribution to *R*_dark_, and that 50% of maintenance costs are attributable to protein turnover (Bathe et al. 2023). When studying protein synthesis and turnover costs in fully expanded, mature leaves that do not have any *R*_dark_ attributable to growth, it has been found, rather astoundingly, that ∼30% of all hexoses consumed due to protein turnover is needed for 2 closely related proteins, Rubisco and Rubisco activase (Li et al. 2017; Bathe et al. 2023). Rubisco activase regulates Rubisco activity by clearing bound substrates that inhibit the Rubisco active site (Mueller-Cajar et al. 2014). Rubisco and its regulation thus account for 15% of the total energy costs of a mature leaf. Rubisco is therefore an important target not only for photosynthetic capacity and *V*_cmax_, but also for influencing the respiratory energy costs associated with its synthesis and turnover. Interestingly, there is evidence that, with warmer growth temperatures that speed up reaction rates, acclimation involves reducing Rubisco abundance in leaves, thereby enabling the same level of *A*_net_ while presumably reducing the maintenance *R*_dark_ demand (Campbell et al. 2007; Scafaro et al. 2017b). The extent to which Rubisco abundance declines with warmer growth temperatures and how these declines affect respiratory carbon loss in crops needs further exploration. Because alterations in Rubisco abundance will contribute to both photosynthetic carbon gain and respiratory carbon loss, such modifications make it a good candidate for improving crop performance.

## Carbon uptake and use must be practically quantifiable in the field

### Time integration

**Figure 3:**
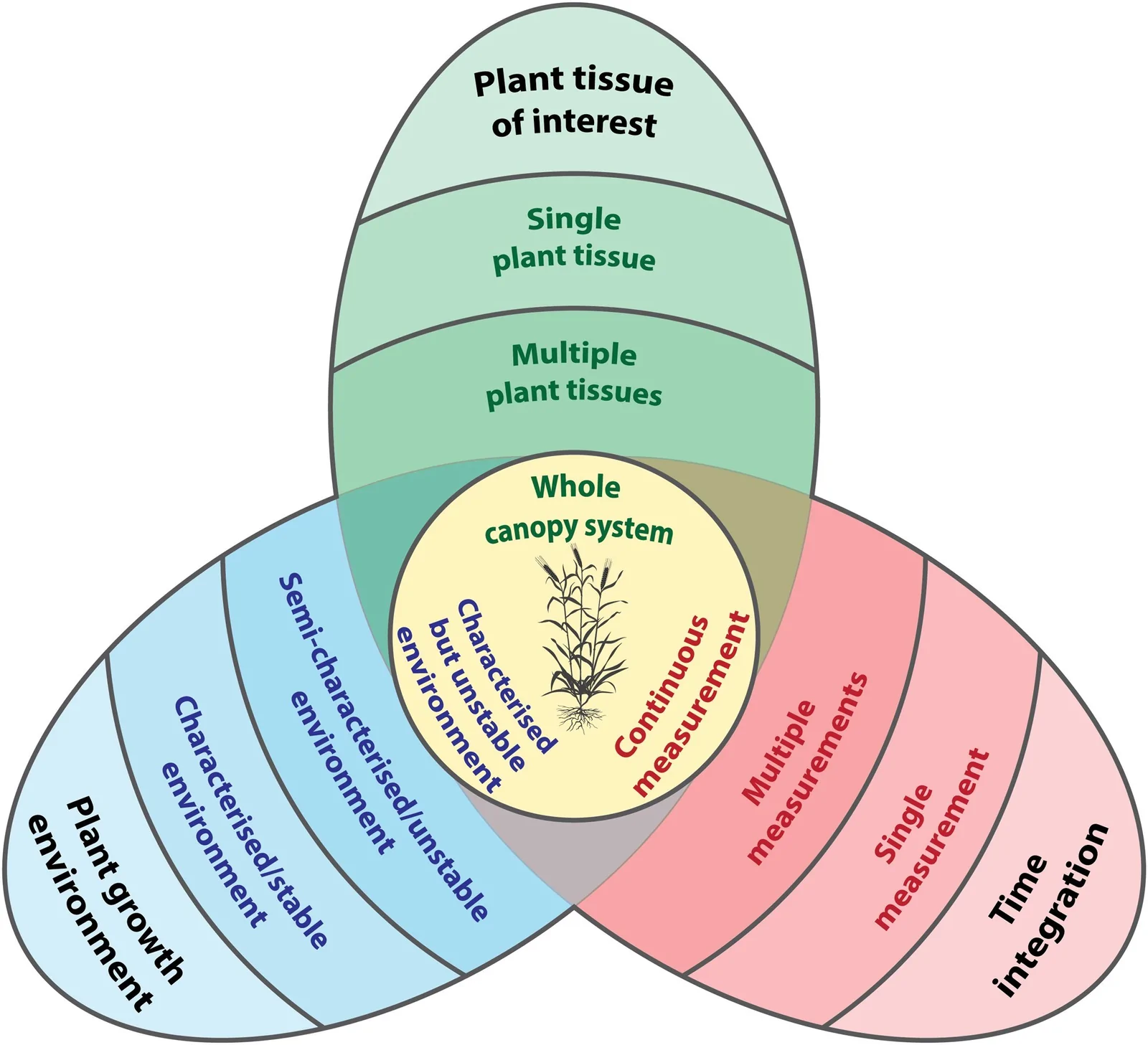
Schematic outlining three perspectives on ways to link crop physiology to improve crop yields. To gain the capacity to predict whole-plant responses, tissue-level knowledge needs to be scaled across multiple tissues, both above and below ground. The understanding of plant physiological responses in well-characterized, stable environments needs to be advanced to predict performance in unstable environments. Here, detailed characterization of field environments will be crucial. This will require reduced reliance on data from remote monitoring stations and greater emphasis on collecting detailed environmental data within crop canopies (as discussed in the Advanced Technology may be the answer to quantifying carbon efficiency in the field section). The separation of environmental influence from genotype in controlling variation in new metabolic traits and minimization of unexplained variance in statistical models, will also be crucial for breeders to invest in targeting new traits. Finally, measurements made at a single time point must be built on to enable continuous monitoring of metabolic traits and plant performance.

More than a single measurement of *A*_net_ or *R*_dark_ is required to correlate carbon metabolism with yield (Fig. 3). Single measurements are generally uncorrelated because they capture only an instant in time for a process that is constantly changing throughout the day, across seasons, and in relation to developmental stage (Zelitch 1982; Fischer et al. 1998; Reynolds et al. 2000; Driever et al. 2014). Light can be sporadic throughout the day and strongly influences the achieved *A*_net,_ irrespective of the underlying photosynthetic capacity (Long et al. 2022). Consideration needs to be given to not only the realized *A*_net_ at any given instant but also the time it takes to reinduce *A*_net_ after a period of low light (potentially 20 min), with photosynthetic induction being dependent on multiple limitations including stomatal reopening, biochemical induction of Rubisco, and chloroplast electron transport (Deans et al. 2019; Yamori et al. 2020).

A further factor to consider is that re-exposure to sunlight (after a period of shading) can dramatically change leaf temperature and increase available photosynthetically active radiation. For example, there are reports of leaf temperature increasing by up to 8 °C within 5 min as a leaf moves from shade to light (Doughty and Goulden 2008). Accounting for leaf temperature is important, as increasing temperature is an exponential driver of higher rates of key *A*_net_ components, at least until the photosynthetic machinery is damaged (Way and Yamori 2014; Scafaro et al. 2023). Similarly, *R*_dark_ rises exponentially with temperature (Atkin and Tjoelker 2003). *R*_dark_ can vary at night independently of temperature, with higher respiratory CO_2_ release observed after sunset, which then falls throughout the night for reasons not yet clearly understood (Bruhn et al. 2022). A further complication is that *R*_dark_ is often measured during the day through dark-adapting leaves (for the convenience of not needing to be observed at night), yet day- and night-xmeasured *R*_dark_ differ significantly due to diel-dependent changes in metabolic activity (Rashid et al. 2020b; Fan et al. 2024; Rana Shahi et al. 2026). Given that *A*_net_ and *R*_dark_ vary throughout the day and night and in response to ever-changing environmental perturbations, accounting for time-dependent changes in carbon flux is necessary to relate them to yield.

### A whole-plant rather than single-tissue approach

While above-ground foliar photosynthesizing tissues can be major determinants of yield, recent studies show that nonfoliar photosynthesizing tissues (eg, spikes, awns and tillers) can contribute up to 50% of crop yield in cereals (Rivera-Amado et al. 2019; AuBuchon-Elder et al. 2020; Molero and Reynolds 2020; Lawson and Milliken 2023). In wheat, Zhang et al. (2020) found that 34% of photosynthetic carbon uptake at anthesis was attributed to leaves. However, the inflorescence spike contributed up to 31% of seed carbon and intercepted up to 30% of the light reaching the canopy. Similarly, in soybean, ∼14% of seed mass was derived from assimilates generated by the seed pod itself, contributing 9% of all canopy photosynthesis (Cho et al. 2023). Even a single leaf cannot be considered physiologically homogeneous. Measuring the 2D surface does not capture cell activity in deeper layers, which experience different levels of irradiance and longer CO_2_ transport pathways (Evans 2009).

Even more so than *A*_net_, *R*_dark_ is not localized to leaves, as all biological tissues must respire. Of crucial importance to the overall carbon economy of crops are rates of *R*_dark_ occurring throughout whole root systems, with 8% to 52% of daily fixed carbon respired back to the atmosphere by roots (Lambers et al. 2002). Influencing this percentage is the ratio of root dry mass to shoot dry mass (ie, root-to-shoot ratio), which in cereals is strongly influenced by the number of nodal roots (Gao and Lynch 2016). The mix of immature to mature roots will also contribute, with specific rates of root respiration declining markedly as roots age (Lambers et al. 2002; Atkin et al. 2009). The extent of mycorrhizal colonization could also be important, as shown by higher in vivo rates of root O_2_ uptake in leeks and plantains colonized by mycorrhizae (Snellgrove et al. 1982; Atkin et al. 2009). However, our understanding of the extent to which root respiration influences the carbon economy of crops under field conditions remains limited. The scarcity of root system data, including root respiration rates and spatial and temporal variation in nutrient and water uptake rates under realistic field conditions, is often attributed to (i) the inherent complex nature of root systems and their interaction with the soil environment; (ii) the technological and economic difficulties with measuring root traits using cumbersome, time-consuming and expensive techniques including soil coring, soil columns, minirhizotrons, or specialized technologi (Azevedo et al. 2011; Smit et al. 2013; Li et al. 2019); and (iii) an assumption that the contribution of roots to final crop yield is small.

### The experimental growth environment

Plant scientists mostly conduct experiments in highly controlled environments, usually involving growth cabinets, growth rooms, or greenhouses. Tight control of growth parameters, such as irradiance, humidity, and temperature, enables experiments to be reproducible. However, experimentation in such controlled settings might not be relevant to what crops experience in the field. Currently, it is not feasible for a controlled environment to fully replicate the field environment, although newer technology is allowing for controlled environments to mimic real-world settings, including dynamic control of light, independent soil temperature control, and temperature regimes that can be programmed to follow the diel patterns observed in nature (Wilson et al. 2019). These developments are moving some controlled growth facilities away from optimal growth conditions and closer toward field simulation (Heuermann et al. 2023). The need to match the field is evident in studies that demonstrate the impact of a highly fluctuating growth environment on key biological traits that contribute to yield. For example, manipulating temperature and irradiance over the day and night cycle alters the genetic regulation of floral genes and flowering time in the model species *Arabidopsis* (and presumably crop species as well), flowering being an important gene-by-environment contributor to yield that would not be evident in a static controlled environment setting (Burghardt et al. 2016). Another important aspect of controlled environments that has become apparent is the abrupt changes from light to dark (square lighting regimes). Square lighting leads to a reduction in sucrose abundance by as much as half within 30 min of darkness, and takes hours to recover, whereas sinusoidal light regimes that mimic dusk do not (Annunziata et al. 2017). Thus, the use of sinusoidal light regimes in controlled settings that match natural lighting at dusk is necessary to remove quite substantial diurnal artifacts on carbon metabolism.

A more direct application of experimenting with crops under defined conditions is the adoption of controlled-environment agriculture (including more innovative vertical farms and plant factories), in which crop production occurs in controlled facilities rather than in the field (Asseng et al. 2020; Engler and Krarti 2021). Indeed, satellite surveys have identified that greenhouses occupied 1.3 million hectares of land in 2019, with the area rising sharply (Tong et al. 2024). As controlled-environment agriculture becomes an ever-increasing share of food production, studying crop physiology in the artificial space of a greenhouse or growth room will become increasingly relevant.

## Advanced technology may be the answer to quantifying carbon efficiency in the field

**Figure 4:**
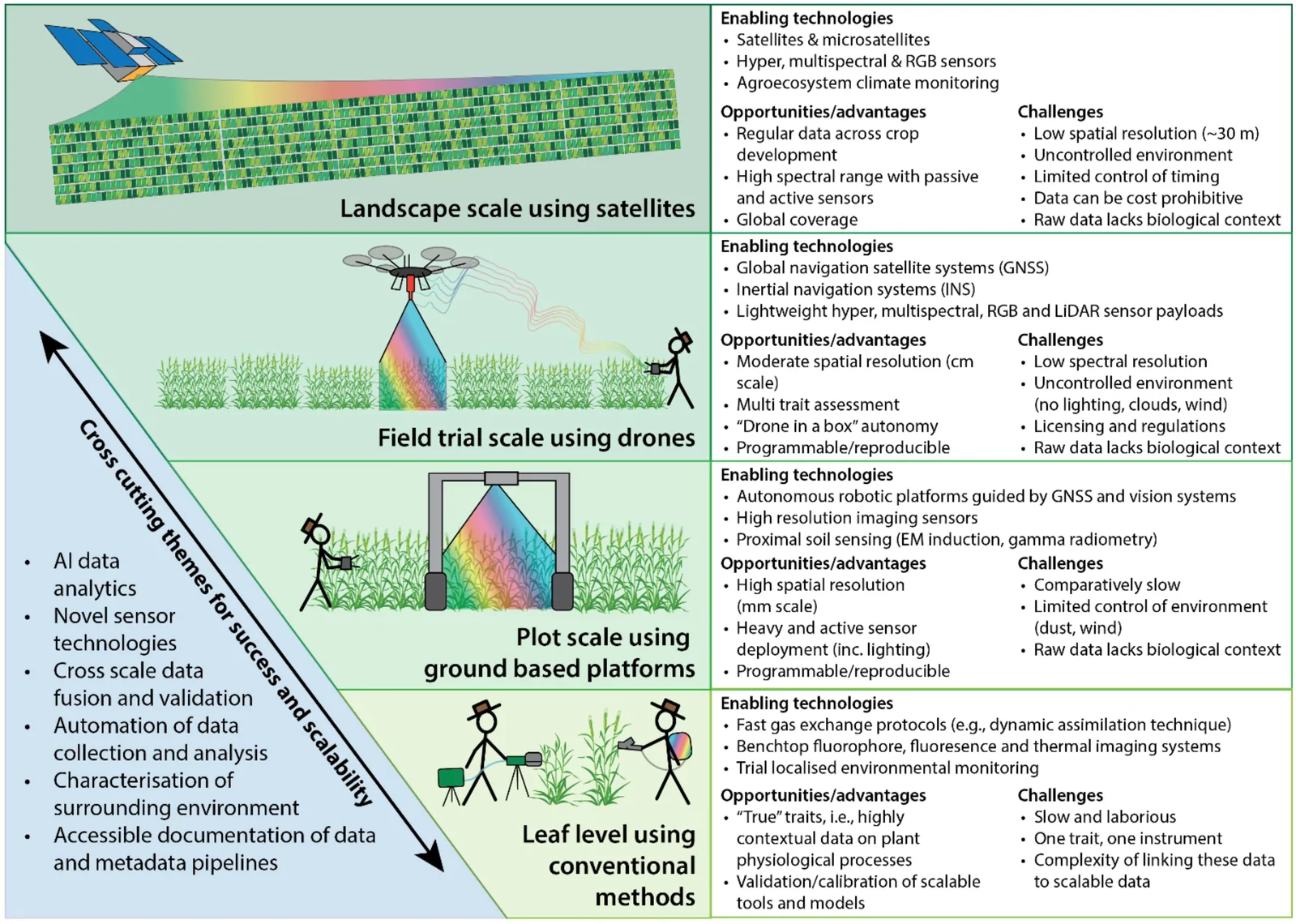
Schematic outlining the range of enabling technologies available to quantify leaf metabolic traits in crops, with a focus on the different spatial (and temporal) scales over which such technologies can be deployed. Key to unlocking the potential of leaf metabolic traits to create a step change in crop productivity will be providing the crop breeding sector with the sensors and data fusion/analysis approaches that will enable variations in rates of leaf photosynthesis and respiration, and associated biochemical traits, to be quantified using sensors that capture the spectra of light that is reflected off canopies; this will involve moving from leaf-level measurements to sensor-based predictions of traits from gantries, drones, and ultimately satellites. However, to be successful, a range of challenges will need to be addressed, such as the lack of control over incident light and sources of reflected light when sensors are not proximal to individual leaves, and the need to fuse multiple forms of complex data, such as canopy structure (eg, using light detection and ranging) and environmental conditions (eg, temperature, soil moisture, vapor pressure deficit).

Technological advancement has long been identified as the golden ticket to enabling the inclusion of complex physiological traits such as *A*_net_ and *R*_dark_ in breeding programs. Indeed, the term “high-throughput phenomics' has existed for decades. Yet photosynthesis and respiration are not easy to characterize using conventional field methods, and, as we have highlighted, to be relevant and useful from a crop improvement perspective, they need to be measured at appropriate scales (Fig. 4). Attempts have been made to improve the throughput of such measurements, including the use of instruments that simultaneously measure multiple samples. For example, fluorophore dyes in conjunction with robotic automation have increased the speed of measuring leaf *R*_dark_ by a factor of 20 (Scafaro et al. 2017a). Similarly, high-throughput systems have been developed to quantify the high temperature-tolerance of photosystem II using dark-adapted fluorescence (Coast et al. 2022; Posch et al. 2022), thermally image canopies (Vialet-Chabrand and Lawson 2020), multiplex leaf gas exchange (Salter et al. 2018), and rapidly quantify CO_2_ response curves of net photosynthesis (Tejera-Nieves et al. 2024). Yet these approaches are still too slow, laborious, or destructive to be adopted by crop physiologists, let alone by plant breeders or farmers. However, recent developments in remotely sensed data collection and analysis technologies now present a tangible opportunity to phenotype photosynthetic and respiratory processes across broad spatial and temporal scales. These tools could translate these complex, previously inaccessible plant physiological processes into selectable traits that the plant breeding community can adopt.

### Advanced sensors

The most promising technological developments for high-throughput plant phenotyping lie in advanced hyperspectral sensors (Fig. 4). Spectral reflectance data collected at the leaf scale using field spectroradiometers and analyzed using multivariate and deep learning models trained using rigorously collected “ground-truthing” data have been shown to deliver robust estimates of *A*_net_, *R*_dark_, and *V*_cmax_ of crops including wheat, soybean, maize, and tobacco (Silva-Pérez et al. 2017; Yendrek et al. 2017; Coast et al. 2019; Fu et al. 2019). This allows for the assessment of these traits in seconds. However, operating at the individual leaf scale and requiring someone to walk around the field site with the instrument still limits data collection to only 1 plant tissue at a time. To overcome this, the next frontier will be the deployment of hyperspectral imaging sensors in crop research, which, rather than single-point spectra on individual leaves, produce 3D data cubes that incorporate spatial and spectral information from the whole canopy (Fig. 4). Advances in high-throughput field-crop phenotyping platforms and predictive phenomics workflows have been comprehensively reviewed [eg, see Araus and Cairns (2014); Yang et al. (2017); Xu and Li (2022); Roth et al. (2025)]. These platforms can now be almost fully autonomous, guided by sophisticated global navigation satellite systems and inertial navigation systems. While these sensors only capture information at 1 point in time, the ability to automate data capture and standardize data through geometric and radiometric calibration procedures enables reproducible, regular data collection throughout a growing season to capture these traits across crop development. The caveat will be ensuring that measurements collected remotely at scale are indicative of physiological processes occurring at the level of individual organs. This will require rigorous scientific research to understand whether these techniques are indeed scalable. There are substantial challenges ahead in recovering the fidelity of leaf-level hyperspectral predictions of complex metabolic traits when reflectance sensors are mounted on aerial platforms. Difficulty arises from the lack of control over incident light (eg, variable exposure of leaf surfaces to solar radiation) and from reflected light sources when sensors are no longer proximal to leaves (Fig. 4).

### Advanced data analysis

For many advanced sensors and technologies to provide contextual information, rather than just raw image files and/or spectra, data processing and analysis pipelines also need to advance. Machine learning-based models can be trained to contextualize such data formats, and the computing resources required to deploy these algorithms at appropriate scales are now readily accessible. Newer deep learning architectures, including semi- and self-supervised learning techniques, are similarly data-hungry; however, they do reduce reliance on large volumes of labeled data (Yan and Wang 2022). Given the laborious and slow nature of phenotyping *A*_net_ and *R*_dark_ at the leaf scale, these new deep learning tools offer great promise. Other plant physiological traits that have shown potential for scaling up with hyperspectral phenotyping and machine learning include stomatal conductance and leaf water potential (Wong et al. 2023). However, this will require extensive collaboration between crop breeders, who possess relevant plant materials, field expertise, and a crop improvement perspective, and plant physiologists, who possess a deep understanding of desired plant physiological processes and the technical expertise to measure them. A prime example is mesophyll conductance (*g*_m_), which has the potential to improve crop carbon fixation by increasing the diffusivity of CO_2_ into chloroplasts (Lundgren and Fleming 2020). *g*_m_ has long been out of reach of crop physiologists due to extreme difficulties in accurate measurement, but machine learning approaches have recently shown promise even for this highly inaccessible trait (Rahimi-Majd et al. 2024). Open sharing of data under FAIR (findable, accessible, interoperable, and reproducible) principles will be essential for deploying these technologies at scale and bridging the information gap between breeders and crop physiologists.

## Conclusions

One of the grand challenges facing society is how to meet the nutritional needs of a growing global population without increasing arable land or causing biodiversity loss. Traditional approaches to crop breeding, developed over thousands of years and accelerated over the past 150 years, have largely ensured that global food requirements are met. Key to this success was the application of crop physiology approaches to understanding the components of yield potential and yield resilience. However, going forward, reliance on past approaches is unlikely to be sufficient, as headwinds from global climate change will limit future gains in crop yields. New approaches will be needed, approaches that take advantage of our increasing understanding of carbon metabolism to control rates of resource capture and resource use in crops. This article provides examples of how advances in technology are enabling tissue-level understanding of plant biology to be scaled to the whole-plant, plot, and landscape levels. When remote sensing of plant growth and performance is combined with detailed characterizations of the environment in which crops are growing, there will be new opportunities to scale tissue-level observations across wide spatial and temporal scales. The latter is particularly crucial, as the ultimate way to connect physiology with yield gains will be to develop methods that enable characterization of complex metabolic processes in the field that are both time- and tissue-specific.

## Data Availability

No new data were generated or analyzed in support of this article.
